# Evaluation of stereotactic radiosurgery conformity indices for 170 target volumes in patients with brain metastases

**DOI:** 10.1120/jacmp.v12i2.3449

**Published:** 2011-02-01

**Authors:** Julia Stanley, Karen Breitman, Peter Dunscombe, David P. Spencer, Harold Lau

**Affiliations:** ^1^ Department of Medical Physics Tom Baker Cancer Centre Calgary AB T2N 4N2 Canada; ^2^ Department of Oncology Tom Baker Cancer Centre Calgary AB T2N 4N2 Canada

**Keywords:** radiosurgery, conformity index, plan quality

## Abstract

A database of clinically approved stereotactic radiosurgery treatment plans was created. One hundred and seventy targets in the database were then retrospectively evaluated using conformity indices suggested by RTOG, SALT‐Lomax and Paddick. Relationships between the three alternative conformity indices were determined. The Paddick index combines the information provided by the RTOG and SALT‐Lomax indices into a single index. The variation in the geometric overlap ratio, which is related to the SALT‐Lomax index, was found to be not clinically relevant for our cohort of patients, and thus the Paddick and RTOG indices can be directly related. It was found that access to a dose volume histogram or dose distribution for a treatment plan renders the RTOG conformity index sufficient for plan quality evaluation.

PACS number: 87.53.Ly

## I. INTRODUCTION

Up to forty percent of patients with cancer have brain metastases sometime during their disease.^(1)^ While brain metastases can arise from any primary cancer, the most common cancers to metastasize to the brain are lung, breast, kidney, colorectal and melanoma.^(2)^ The main treatment options available for brain metastases patients are surgery, whole brain radiotherapy, chemotherapy and stereotactic radiosurgery (SRS).^(3)^ SRS, which was originally developed by the neurosurgeon Lars Leksell,^(4)^ is a form of external beam radiation therapy which aims to precisely deliver a high dose to the target volume in a single fraction and concurrently spare surrounding healthy tissue. SRS is appropriate for brain metastases patients who have fewer than five brain metastases all of which have a diameter of less than 4 cm.^(5)^ SRS treatment plans require a high degree of conformity to minimize damage to the tissues that surround the target area. Clinically, this conformity is evaluated using a combination of visual inspection of the plan and evaluation of the dose‐volume histograms (DVHs). However, the plan quality information provided by such methods cannot be easily quantified to allow for comparison between plans. To facilitate comparison and to evaluate compliance with clinical trial protocols, a number of plan quality metrics has been proposed. One type of plan quality metric is geometry‐based. Each index takes factors such as the overlap and size of dose and target volumes into account. In this study, a group of 170 SRS targets has been evaluated using the Radiation Therapy Oncology Group (RTOG) conformity index, the conformity index proposed by Paddick and the conformity index proposed by Lomax and Scheib, with a view to determining if the information provided by different conformity indices is either congruent or independent.

## II. MATERIALS AND METHODS

### A. Database

A database of patient information for 170 targets was constructed from 105 patients with brain metastases treated at our institution from November 2004 to December 2009. The database information was obtained from plans created using the clinically implemented BrainScan 5.31 (BrainLAB Inc., Germany) planning software. All dose‐volume histogram (DVH) calculations were performed using a grid resolution of 0.5 mm^3^. The distribution of target sizes is given in Table 1 and patient characteristics are given in Table 2.

**Table 1:** Distribution of target volumes.

*Target Size*	*Number of Targets*
0 to ≤1 cc	79
1 to ≤2 cc	27
2 to ≤3 cc	21
3 to ≤4 cc	14
4 to ≤5 cc	5
5 to ≤10 cc	14
>10 cc	10

**Table 2:** Distribution of characteristics of the 105 patients in the database.

*Patient Characteristic*	*Distribution*	
Age Median (Range)	60 (35−81)	
Sex Male:Female	39:66	
Primary Tumor Site	Breast	33
	Lung	46
	Renal	5
	Melanoma	9
	Other	12
Dose Prescription to 80% isodose for 170 targets	≤15 Gy	22
	15.1−≤18 Gy	123
	>18 Gy	25
Number of targets per treatment	One target	72
(8 patients treated twice)	Two targets	27
	Three targets	12
	>Three targets	2

### B. RTOG Criteria

The Radiation Therapy Oncology Group (RTOG) proposed three widely used metrics that can be employed to describe the quality of stereotactic radiosurgery (SRS) plans.^(6)^ The first metric is the conformity index, CIRTOG, which is given by

(1) $$CI_{RTOG}=\frac{V_{RI}}{TV}$$

where $V_{\text{RI}}$ is the volume encompassed by the prescription isodose and *TV* is the target volume.^(3)^ Each target in the database had been contoured by an oncologist and its target volume was then subsequently calculated by the planning software, BrainScan through its dose‐volume histogram function. The corresponding $V_{\text{RI}}$ was determined by constructing a sphere encompassing the 50% isodose line and then determining the volume of the sphere that received the prescription isodose. This strategy was employed to reduce the calculation time. The RTOG defines three categories of conformity index protocol compliance. Plans with a conformity index value between 1.0 and 2.0 are classified as not deviating from RTOG protocol; plans with a conformity index value between 2.0 and 2.5 or between 0.9 and 1.0 are classified as having minor deviations; plans with a conformity index value greater than 2.5 or less than 0.9 are classified as having major deviations.^(6)^

The second metric developed by the RTOG to evaluate SRS plans is quality of coverage, Q. This value is found using the expression:

(2) $$Q=\frac{I_{\min}}{RI}$$

where $I_{\text{min}}$ is the minimum dose given to the target and *RI* is the prescription isodose.^(7)^ Both these values were found using BrainLAB's histogram function for each target in the database. Plans where the 90% isodose covers the target do not deviate from protocol, plans where the 80% isodose covers the target are classified as having a minor deviation, and plans where the 80% isodose line does not fully cover the target are classified as having a major deviation.

The third metric proposed by the RTOG to evaluate SRS plan quality is the homogeneity index, HI. This index is defined by the equation

(3) $$HI=\frac{I_{\max}}{RI}$$

where $I_{\text{max}}$ is the maximum dose in the target and *RI* is the prescription isodose.^(7)^ Plans with a homogeneity index less than or equal to 2 are said to not deviate from protocol. Minor deviations result when the homogeneity index is between 2 and 2.5, and major deviations result when the value is greater than 2.5.6

### C. Alternative conformity indices

In 2000 Paddick^(8)^ proposed an alternative conformity index with the goal of providing an objective method for comparing plan quality and eliminating “false scores” provided by the RTOG index. The proposed index builds on the criticism of the RTOG index that the overlap of the volume receiving the prescription isodose and the target volume is not accounted for. This new conformity index, ${\text{CI}}_{\text{Paddick}}$ is defined as:

(4) $$CI_{Paddick}=\frac{TV_{PIV}^{2}}{\left(TV\times V_{RI}\right)}$$

where *TV* is the target volume, TV*PIV* is the target volume covered by the prescription isodose and V*RI* is the total volume covered by the prescription isodose.^(8)^ All three of these parameters were found using the dose‐volume histogram calculated by the treatment planning system. This index has an ideal value of one and plan quality decreases with decreasing index value.

This new conformity index can also be rewritten in terms of the RTOG index. The resulting relationship is:

(5) $${\text{CI}}_{\text{Paddick}}=\frac{TV_{PIV}^{\;2}}{TV^{2}\times CI_{RTOG}}$$

Thus, the Paddick and RTOG indices are inversely related. The geometric overlap ratio $\frac{{\text{TV}}_{\text{PIV}}^{\;2}}{{\text{TV}}^{2}}$ provides a correction term to the RTOG index by accounting for the geometric overlap of the target volume and treated volume.

Another alternative conformity index to the RTOG conformity index was proposed by Lomax and Scheib.^(9)^ Their index is a modification of the stereotactic plan quality criterion initially proposed by the Saint‐Anne, Lariboisiere, Tenon (SALT) group for arteriovenous malformations. Lomax and Scheib's modified index, ${\text{CI}}_{\text{Lomax}}$ is given by:

(6) $$CI_{Lomax}=\frac{TV_{PIV}}{TV}$$

where ${\text{TV}}_{\text{PIV}}$ is the target volume covered by the prescription isodose and *TV* is the target volume.^(7)^ This new index, in effect, shows the proportion of the target volume that receives at minimum the prescription dose. This conformity index can range from 0 to an optimum value of 1 when the target volume in its entirety receives at least the prescribed dose. This index is the square root of the geometric overlap ratio that is used in the Paddick index.

## III. RESULTS

### A. RTOG

**Figure 1:**
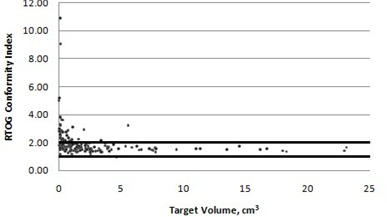
RTOG conformity index versus target volume. Points that fall within the horizontal lines at 1 and 2 are defined as being within protocol.

A graph of RTOG conformity index versus target volume was constructed for the 170 brain metastases in the database and can be seen in Fig. 1. In general, plans with smaller target volumes had larger conformity indices, whereas the conformity index was relatively constant for plans with larger target volumes. The maximum conformity index value of 10.89 occurred for a plan with a target volume of 0.12 cc. For the target volumes larger than 1 cm^3^ the average conformity index value was found to be 1.65. For plans with target volumes smaller than 1 cm^3^ the mean conformity index value was 2.35. The overall conformity index average was found to be 1.988. The comparable value found by Lomax et al.^(9)^ was 1.24.

Of the 170 targets examined, 10.00% (17) had minor deviations and 13.53% (23) had major deviations (counting conformity indices of 2.0 as nondeviations). Of the targets with major deviations from protocol, 20 out of 23 or 87% had target volumes smaller than 1 cm^3^.

**Figure 2:**
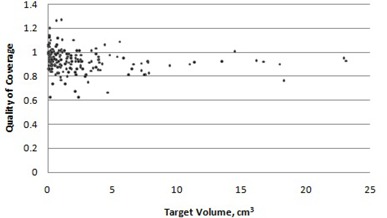
RTOG quality of coverage versus target volume.

A graph of RTOG quality of coverage versus target volume can be seen in Fig. 2. The minimum quality of coverage value was 0.63 and the maximum value was 1.27. The average and median quality of coverage values were 0.930 and 0.925, respectively.

**Figure 3:**
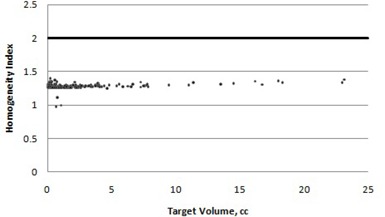
RTOG homogeneity index versus target volume. Points that fall below the horizontal line are defined as being within protocol.

A plot of homogeneity index versus target volume is given in Fig. 3. All 170 targets had a homogeneity index that was within protocol values.

### B. Comparison of alternative indices

**Figure 4:**
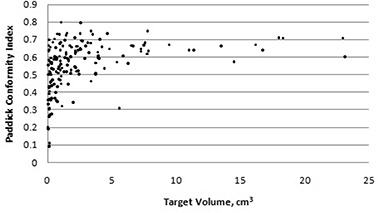
Paddick conformity index versus target volume.

The conformity index suggested by Paddick was plotted against target volume for the 170 target volumes in the database, as shown in Fig. 4.

No acceptable limits for the new conformity index were defined by Paddick. Once again, the conformity index is not independent of target volume: the average conformity for all the targets was 55.6%, whereas the average conformity for targets smaller than 1 cm^3^ was 48.7%.

**Figure 5:**
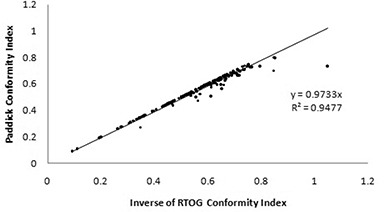
Relationship between Paddick and RTOG conformity indices.

Next, the Paddick conformity index was compared to the RTOG index by plotting the Paddick conformity index values against the inverse of the RTOG conformity values, as shown in Fig. 5.

The data were fit with the equation:

(7) $$CI_{Paddick}=\frac{0.9733}{CI_{RTOG}}$$

**Figure 6:**
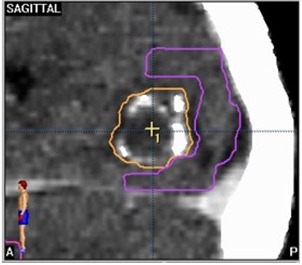
An example where the geometric overlap ratio is less than one due to an adjacent critical structure. The target volume is outlined in orange and the occipital cortex is outlined in purple.

This fit had an $R^{2}$ value of 0.9477. However, since the Paddick conformity index has a maximum value of 100%, this equation is only valid over the range of values where the Paddick conformity index is between 0 and 100%. Outliers occur when the geometric overlap ratio, $\frac{TV_{\text{PIV}}^{\;2}}{{\text{TV}}^{2}}$, is less than one. This ratio was also calculated for all targets and the resulting data are presented in Table 3. The vast majority of targets (89%) had a geometric overlap ratio greater than 0.95. Equations (5) and (7) can be combined to show that the geometric overlap ratio for the dataset has a value of 0.9733. There was one target that had a geometric overlap ratio less than 0.75; this target was situated directly adjacent to a critical structure – the occipital cortex, as seen in Fig. 6.

**Table 3:** Geometric ratio overlap occurrences.

*Ratio Value*	*Number of Occurrences*
<0.75	1
0.75 to 0.8	1
0.8 to 0.85	3
0.85 to 0.9	2
0.9 to 0.95	12
>0.95	151

**Figure 7:**
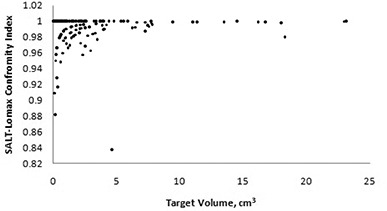
SALT‐Lomax conformity index versus target volume.

A plot of the SALT‐Lomax conformity index for the database is given in Fig. 7. Interestingly, a total of 56% or 95 of the 170 targets evaluated had a perfect SALT‐Lomax conformity index value when rounded to two decimal places. All the examined targets had a SALT‐Lomax conformity index value greater than 0.8.

## IV. DISCUSSION

The RTOG overall conformity index average for the 170 targets was found to be 1.988. The comparable value found by Lomax and Scheib^(9)^ was 1.24. One hundred and thirty of the targets met the conformity index values to be classified as “per protocol” by the RTOG. The majority of targets that are classified as having major deviations from protocol (87%) have volumes smaller than 1 cm^3^. Lomax and Scheib^(9)^ suggested that, while for larger target volumes the RTOG conformity index is target volume independent, for target volumes smaller than 1 cm^3^ the conformity index is influenced by target volume. This assertion is supported by our dataset. Knöös et al.^(10)^ suggested that the influence of target size is due to the fact that when the PTV is small, a small change in absolute volume will translate into a large relative change.

The examination of the same targets’ SALT‐Lomax conformity index values showed that 56% of the targets that were approved for treatment resulted in ideal conformity index values. There was only one target which had a conformity index value not greater than 0.8; this target also had a major deviation from protocol classification when evaluated using the RTOG criteria.

When the conformity index suggested by Paddick was used to evaluate the set of targets, the average conformity was 55.6%. This conformity index followed a similar pattern to the RTOG conformity index: the conformity worsened for targets smaller than 1 cm^3^. Wu et al.^(11)^ also noted that the Paddick conformity index behaved similarly to the RTOG conformity index with respect to small target volumes.

A number of alternative conformity indices have been suggested over the past decade. The conformity index put forward by the RTOG has been widely in use since its introduction in 1993. This index has been criticized for its lack of consideration of the spatial overlap of the target and treated volumes. Subsequently, modifications to the RTOG conformity index have been proposed by groups such as Paddick to account for spatial overlap. This overlap was calculated using the geometric overlap ratio. In 89% of the examined targets the geometric overlap ratio was greater than 0.95, which suggests that in clinically used plans the information provided by this ratio is not critical for evaluating plan quality provided a dose distribution is available for visual inspection. Most of the time, routine clinical planning results in geometrically overlapping target volumes and prescription volumes. When plans deviated from this norm, the lower ratio value can be accounted for by extenuating circumstances such as proximity to critical structures. It is expected that when a target is in close vicinity to a critical structure, the geometric overlap ratio will systematically deviate from the ideal value of one due to constraints arising from the need to spare the structure. Our data, however, suggest that the conformity index corrected for geometric overlap provides the same plan quality information as does the RTOG conformity index for the majority of targets. While theoretically it is possible for the RTOG conformity index to give a “perfect score” to a plan where the target volume and treated volume do not overlap, this situation does not routinely occur in clinically approved plans.

## V. CONCLUSIONS

The stereotactic plans approved for clinical use at the Tom Baker Cancer Centre were initially created using an amalgamation of information derived from planner experience, visual inspection and dose‐volume histograms. Seventy‐six percent of the planned targets retrospectively were classified as “per protocol” for the RTOG conformity index; 56% of these targets had ideal SALT‐Lomax conformity index values, and the average Paddick conformity was also 56%. The Paddick index incorporates information provided by both the SALT‐Lomax and RTOG conformity indices. In the vast majority of the clinical targets examined, the target volume and treated volume overlap and, therefore, the Paddick index could be reduced to the inverse of the RTOG index. If a review of the dose‐volume histogram or dose distributions can be performed, the Paddick index effectually provides the same information as the RTOG index.
